# Semiquantitative Approach to Amyloid Positron Emission Tomography Interpretation in Clinical Practice

**DOI:** 10.1055/s-0042-1757290

**Published:** 2022-10-28

**Authors:** Ana M. Franceschi, David R. Petrover, Luca Giliberto, Sean A. P. Clouston, Marc L. Gordon

**Affiliations:** 1 Neuroradiology Section, Department of Radiology, Lenox Hill Hospital, Donald and Barbara Zucker School of Medicine at Hofstra/Northwell, New York, New York, United States; 2 Institute for Neurology and Neurosurgery, Donald and Barbara Zucker School of Medicine at Hofstra/Northwell, Manhasset, New York, United States; 3 Litwin-Zucker Research Center, Feinstein Institutes for Medical Research, Northwell Health, New York, United States; 4 Department of Family, Population and Preventative Medicine and Program in Public Health, Renaissance School of Medicine at Stony Brook University, Stony Brook, New York, United States; 5 Departments of Neurology and Psychiatry, Donald and Barbara Zucker School of Medicine at Hofstra/Northwell, New York, United States

**Keywords:** β-amyloid, ^18^ F-florbetaben, PET/CT, brain PET, Alzheimer's disease

## Abstract

**Objective** Amyloid positron emission tomography (PET) plays a vital role in the in vivo detection of β-amyloid accumulation in Alzheimer's disease. Increasingly, trainees and infrequent readers are relying on semiquantitative analyses to support clinical diagnostic efforts. Our objective was to determine if the visual assessment of amyloid PET may be facilitated by relying on semiquantitative analysis.

**Methods** We conducted a retrospective review of [ ^18^ F]-florbetaben PET/computed tomographies (CTs) from 2016 to 2018. Visual interpretation to determine Aβ+ status was conducted by two readers blinded to each other's interpretation. Scans were then post-processed utilizing the MIMneuro software, which generated regional-based semiquantitative Z-scores indicating cortical Aβ-burden.

**Results** Of 167 [ ^18^ F]-florbetaben PET/CTs, 92/167 (reader-1) and 101/167 (reader-2) were positive for amyloid deposition (agreement = 92.2%, κ = 0.84). Additional nine scans were identified as possible Aβ-positive based solely on semiquantitative analyses. Largest semiquantitative differences were identified in the left frontal lobe (Z = 7.74 in Aβ + ; 0.50 in Aβ − ). All unilateral regions showed large statistically significant differences in Aβ-burden ( *P* ≤ 2.08E-28). Semiquantitative scores were highly sensitive to Aβ+ status and accurate in their ability to identify amyloid positivity, defined as a positive scan by both readers (AUC ≥ 0.90 [0.79–1.00]). Spread analyses suggested that amyloid deposition was most severe in the left posterior cingulate gyrus. The largest differences between Aβ +/Aβ− were in the left frontal lobe. Analyses using region-specific cutoffs indicated that the presence of amyloid in the temporal and anterior cingulate cortex, while exhibiting relatively low Z-scores, was most common.

**Conclusion** Visual assessment and semiquantitative analysis provide highly congruent results, thereby enhancing reader confidence and improving scan interpretation. This is particularly relevant, given recent advances in amyloid-targeting disease-modifying therapeutics.

## Introduction

The most common cause of neurodegenerative dementia is Alzheimer's disease (AD), accounting for 60–80% of all dementias and affecting over 6 million people in the USA and 50 million people worldwide. AD is a leading cause of mortality in the elderly (sixth-leading cause of death in the United States and the fifth-leading cause of death among Americans aged 65 years and older) and, with increasing longevity, is projected to impact up to 13.8 million people in the USA by 2060 with an associated significant rise in healthcare cost, which is projected to exceed $1 trillion by 2050. 1 2 3 Histopathologically, the AD cascade, originally described by Alois Alzheimer in 1907, is characterized by brain infiltration of two amyloid-β (Aβ) isoforms ( *Aβ _1-42_* and *Aβ _1-40_* ), leading to Aβ deposition and the formation of diffuse and dense-core Aβ plaques with concomitant phosphorylation of tau protein and accumulation of neurofibrillary tangles and neurodegeneration. 4 5 6 Amyloid-β in the human brain follows a distinct sequence in which the regions are hierarchically involved, beginning in the neocortex and spreading anterogradely into regions that receive neuronal projections from regions already exhibiting cortical Aβ burden, as described by Thal et al in 2002. 7 Of note, amyloid deposition precedes cognitive changes and tau deposition although the relationship between amyloid, tau, and clinical progression is complex and still needs to be fully elucidated. 8 9

There is intense interest in minimally invasive neuroimaging biomarkers in neurodegenerative disease definitions and for use in clinical trials, particularly in the setting of novel disease-modifying therapies, where accurate confirmation of AD pathology is paramount. 10 In 2016, Jack et al outlined a novel system as an unbiased descriptive classification scheme for research purposes when characterizing patients presenting with cognitive impairment, 11 12 classifying key biomarkers into three binary categories: “A” refers to the value of total β-amyloid biomarker (amyloid positron emission tomography [PET], or cerebrospinal fluid [CSF] Aβ42 can be used); “T,” to the value of a tau biomarker (tau PET, CSF p-tau, or recently plasma p-tau); and “N,” to a biomarker of neurodegeneration or neuronal injury ( ^18^ F-fluorodeoxyglucose–PET, structural magnetic resonance imaging [MRI], CSF total tau, or plasma neurofilament light chain). Each biomarker category is rated as positive or negative and staging can be accomplished for research purposes by the presence/absence of these biomarkers. Given its independent approach to categorizing multidomain biomarker findings at the individual level, without specifying disease labels per se, it is ideally suited as a research framework given the lack of consensus on terminology across the spectrum of cognitive aging and impairment. In fact, the A/T/N classification has been endorsed by the National Institute on Aging and the Alzheimer's Association (NIA-AA) for implementation in the research and clinical trial setting. 12 13

In the characterization of AD for research, amyloid PET plays a vital role in in vivo detection of β-amyloid (Aβ) accumulation in patients presenting with cognitive impairment, and this is taking on a new dimension with the recent Federal Drug Administration's (FDA) approval of the first potentially disease-modifying therapy in the treatment of AD, 14 the amyloid-targeting monoclonal antibody aducanumab (trade name: Aduhelm) in June 2021. Given the first-of-its-kind treatment for AD, trainees and infrequent neuroradiological readers will be required to interpret these studies in a clinical setting, and their interpretations will have a direct impact on patient management. Therefore, semiquantitative tools available in routine clinical practice are becoming increasingly important to support diagnostic efforts. However, there are limited data assessing the correspondence between visual assessment and semiquantitative analysis in the evaluation of cortical amyloid burden. 15 16 17 In this study, we retrospectively reviewed ^18^ F-florbetaben PET/CT studies acquired at our institution as part of the Imaging Dementia-Evidence for Amyloid Scanning (IDEAS) study 18 to determine if the qualitative assessment of amyloid PET scans may be facilitated by relying on results from the semiquantitative analysis.

## Methods

*Ethics:* This study received local institutional review board (IRB) approval. The IRB waived the need for written informed consent, given the retrospective nature of the study.

### Patient Selection

Subjects were referred from memory clinics for ^18^ F-florbetaben PET/CT as part of the IDEAS study from June 2016 to May 2018. Eligible patients were Medicare beneficiaries age 65 years or older, English or Spanish speaking, with a diagnosis of mild cognitive impairment (MCI) or dementia established by a dementia specialist within the past 24 months. All subjects were required to have completed a comprehensive diagnostic assessment, including global cognition assessed via the Mini-Mental State Examination (range, 0 [worst] to 30 [best]) or Montreal Cognitive Assessment (range, 0 [worst] to 30 [best]) at the time of enrollment, laboratory testing within the past 12 months, and head CT or MRI within the past 24 months. Patients were further required to meet appropriate use criteria for amyloid PET 19 : (1) the etiologic cause of cognitive impairment remained uncertain after a comprehensive evaluation by a dementia specialist, (2) Alzheimer's disease was a diagnostic consideration, and (3) knowledge of amyloid PET status was expected to alter diagnosis and management. Patients were excluded if amyloid status was already known based on prior PET or CSF analysis or if learning amyloid status could, in the opinion of the specialist, cause significant psychological harm.

### Image Acquisition

Amyloid PET was completed within 30 days of the pre-PET assessment following published practice guidelines. 20 Subjects received 300 MBq (8.1 mCi) of ^18^ F-florbetabem intravenously and were placed in a quiet room during the tracer uptake period. Approximately 90 to 120 min after radiopharmaceutical injection, patients were scanned on a GE Discovery 710 PET/CT scanner. Transmission data (head CT) was acquired for approximately 2 min and parameters were as follows: kVp 120; mA 95; helical scanning; rotation = 0.8 mm/rot; and pitch = 1.375:1. Emission data (brain PET) was acquired for approximately 20 min (static acquisition with dynamic replay in 10 frames at 2 min/frame). Images were reconstructed using the VUE Point FX reconstruction with 32 subsets and two iterations; z-axis filter = “heavy” and 4.0 mm FWHM Gaussian post-filtering. Quantitative corrections included attenuation, scatter, random, detector efficiency, decay, and deadtime. Acquired PET data were scaled to injected dose and body weight to produce standardized uptake value (SUV) images.

### Image Analysis

Visual interpretation to determine Aβ+ status was conducted by two independent readers blinded to each other's interpretation. One of the readers (DP) was a trainee with 1 year of experience, the other (AMF) was a board-certified neuroradiologist with dedicated PET/MRI training and 5 years of clinical and research expertise in brain PET. Studies were classified as either positive (1) or negative (0) for increased cortical Aβ burden. After qualitative reads were completed, scans were post-processed utilizing MIM-Neuro version 7.1.6 (MIM Software, Inc., Cleveland, Ohio, United States). MIMNeuro is a commercially available program that aids in the analysis and post-processing of PET and single-photon emission computed tomography (SPECT) studies. It performs voxel- and region-based comparison of radiotracer uptake between its database of normal studies and a given study with the aid of built-in anatomic atlases. The differences in radiotracer uptake are represented with a Z-score of standard deviation from normal, or the SUV ratio. For our study, MIMNeuro generated regional-based semiquantitative Z-scores, indicating cortical amyloid burden following industry standards including using the whole cerebellum as the reference region. Z-score values were reported for 12 brain regions total: right and left posterior cingulate gyrus, right and left precuneus, right and left inferior medial frontal gyrus, right and left anterior cingulate gyrus, right and left lateral temporal lobe, and right and left superior parietal lobule.

### Statistical Analysis

Descriptive characteristics are provided using averages, standard deviations, and percentiles. Examples of positive and negative scans are provided. Reader agreement was reported using the Kappa statistic (κ), and overall levels of agreement were also reported. Unilateral regional Z-scores and differences between amyloid-positive and amyloid-negative scans were reported. The area under the receiver-operating curve (AUC) was reported as a measure of overall predictive power for semi-quantitative Z-scores to identify amyloid-positive individuals. Regional spread of amyloid was reported using a number of regions exceeding 2.575 and 3.3 standard deviations above normal ( *p* = 0.01 and *p* = 0.001, respectively). Patients were presented in rank order from the highest to the lowest mean amyloid positivity scores across unilateral regions (organized sequentially with left on top and right below). A two-tailed α = 0.05 was used to determine statistical significance.

## Results

**Fig. 1:**
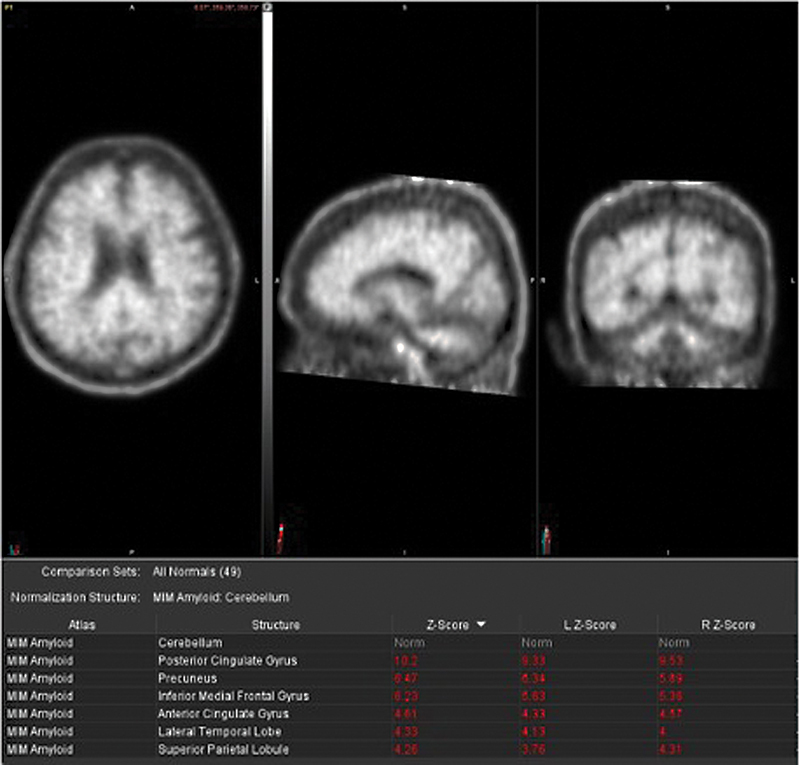
( **A** ) Positive ^18^ F-florbetaben PET/CT with diffuse tracer uptake in the cerebral cortex. ( **B** ) Semiquantitative analysis using the z-scores calculated in comparison to age-matched normal controls reveals significantly increased z-score values in analyzed brain regions including in the posterior cingulate gyrus, precuneus, inferior medial frontal gyrus, anterior cingulate gyrus, lateral temporal lobe, and superior parietal lobule (reference region: cerebellum).

**Fig. 2:**
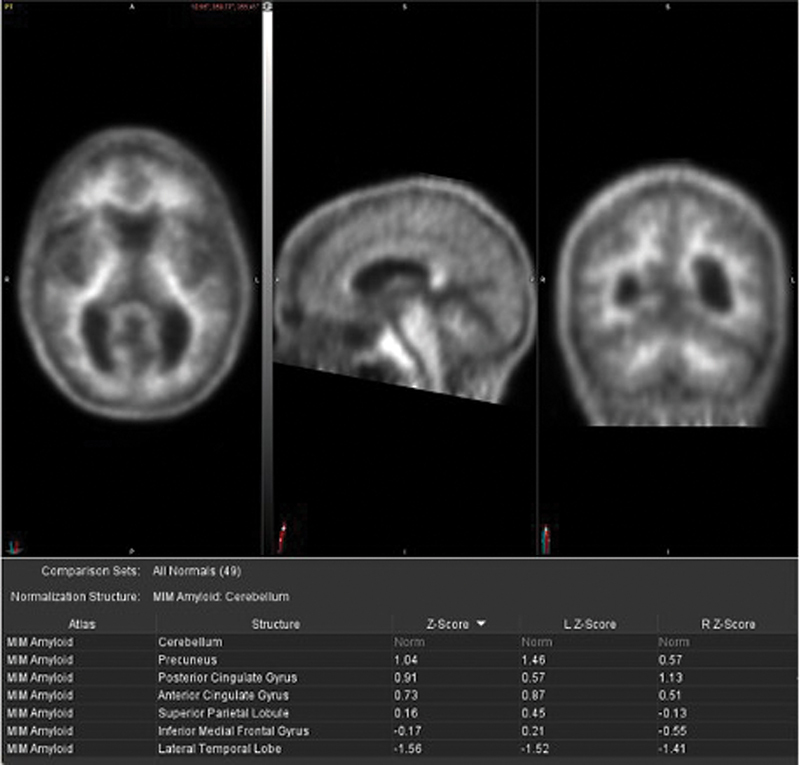
( **A** ) Negative ^18^ F-florbetaben PET/CT with tracer uptake only in the cerebral white matter, and no evidence of amyloid burden in the cerebral cortex. ( **B** ) Semiquantitative analysis using the z-scores calculated in comparison to age-matched normal controls reveals no significantly increased z-score values in analyzed brain regions including in the posterior cingulate gyrus, precuneus, inferior medial frontal gyrus, anterior cingulate gyrus, lateral temporal lobe, and superior parietal lobule (reference region: cerebellum).

A total of 167 subjects (83 females, 84 males; mean age 76.1 ± 6.8 years) underwent ^18^ F-florbetaben PET/CT. Of these cases, 92/167 (reader 1) and 101/167 scans (reader 2) scans were considered positive for amyloid deposition (agreement = 92.2%, κ = 0.84). Figs. 1 and 2 demonstrate an example of a positive and negative amyloid brain PET, respectively, upon visual analysis. Notably, 12 of the 13 cases with visually discordant results were deemed positive in some or all regions by semiquantitative analysis. Upon repeat (unblinded) visual assessment, the readers sided with semiquantitative results in all but one case (92%). Furthermore, 4 of these 13 cases (31%) demonstrated advanced atrophy on structural imaging, which oftentimes poses a diagnostic challenge, especially for qualitative analysis.

An additional nine scans were subsequently identified as possible amyloid-positive based solely on semiquantitative analyses. The largest semiquantitative differences were identified in the left frontal lobe (Z = 7.74 in Aβ+ vs. 0.50 in Aβ− subjects), as depicted in Table 1 . All unilateral regions showed statistically significant differences in Aβ burden ( *p* ≤ 2.08E-28 in all cases). Semiquantitative scores were highly sensitive to Aβ+ status and highly accurate in their ability to identify amyloid-positive patients ( Table 2 ), defined as a positive scan by both readers (AUC ≥ 0.90 [0.79–1.00]). Of the nine cases in which both reviewers evaluated the case to be negative and semiquantitative analysis deemed the case positive, advanced atrophy was present in three cases (33%). In addition, limited regional-only amyloid deposition was present in five of the nine cases (55%), which were positive solely by semiquantitative analysis.

**Table 1:** Regional Z-score differences between individuals determined to be visually positive versus negative on qualitative analysis

Region	Mean Z-score negative	SD negative	Mean Z-score positive	SD positive	Diff.	Area under the receiver-operating curve (95% C.I.)	*p* -Value
Left PCG	1.81	2.53	8.17	2.29	6.36	0.96 (0.93–1.00)	2.42E-35
Right PCG	1.99	2.51	8.09	2.31	6.11	0.96 (0.93–0.99)	7.16E-34
Left precuneus	1.07	2.07	8.22	2.43	7.15	0.98 (0.95–1.00)	1.10E-46
Right precuneus	1.09	1.93	7.18	2.17	6.09	0.97 (0.95–1.00)	3.73E-43
Left frontal	0.50	2.29	7.74	2.68	7.25	0.98 (0.95–1.00)	1.54E-42
Right frontal	0.36	2.36	7.03	2.48	6.67	0.97 (0.94–1.00)	5.34E-39
Left parietal	0.86	1.52	5.36	1.96	4.50	0.96 (0.94–0.99)	3.17E-37
Right parietal	0.79	1.35	4.33	1.62	3.54	0.94 (0.91–0.98)	2.17E-33
Left temporal	0.25	1.54	5.52	2.20	5.27	0.98 (0.96–1.00)	2.14E-41
Right temporal	0.37	1.41	4.86	2.10	4.50	0.96 (0.93–0.99)	1.01E-36
Left ACG	−0.08	2.00	4.34	2.05	4.42	0.94 (0.91–0.98)	4.38E-29
Right ACG	–0.13	2.09	4.40	2.13	4.53	0.90 (0.79–1.00)	2.08E-28

Abbreviations: ACG: anterior cingulate gyrus; AUC: area under the receiver-operating curve; Diff.: difference between amyloid+ and amyloid- participants; PCG: posterior cingulate gyrus; 95% CI: 95% confidence interval.

**Table 2:** Cut points derived for each cortical region along with sensitivity and specificity at the optimal cut point

Regional Z-scores	Suggested cutoffs	Sensitivity at cutoff	Specificity at cutoff
Left PCG	4.63	0.94	0.93
Right PCG	5.38	0.90	0.94
Left precuneus	3.87	0.97	0.96
Right precuneus	4.58	0.90	0.96
Left frontal	3.82	0.96	0.96
Right frontal	3.24	0.96	0.94
Left parietal	3.20	0.85	0.96
Right parietal	1.69	0.94	0.82
Left temporal	2.21	0.95	0.93
Right temporal	2.09	0.95	0.90
Left ACG	1.65	0.90	0.87
Right ACG	1.44	0.94	0.83

Abbreviations: ACG: anterior cingulate gyrus; PCG: posterior cingulate gyrus.

**Fig. 3:**
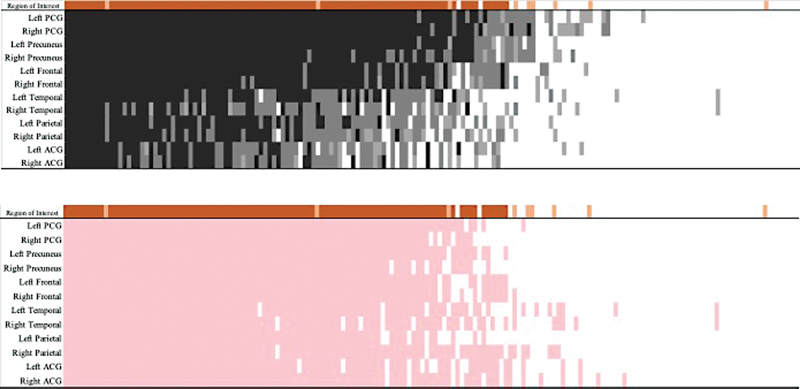
Regional amyloid positivity analysis across patients. Patients are presented in rank order from the highest to the lowest mean amyloid positivity scores across unilateral regions (organized sequentially with left on top and right below). Regions are ordered from most affected ( *top* ) to least affected. Qualitative analysis status is shown using orange bars along the top, with two positive reads indicated by the presence of a dark orange bar and a single positive read indicated by a lighter orange bar. Panel A: Person-region observations are colored based on deviation from expectations (2.58 SDs are light gray, 3.3 SDs are dark gray, 5 SDs are charcoal). Panel B: Person-region observations are colored based on whether that observation cleared the estimated region-specific cutoffs noted in Table 2 . ACG: anterior cingulate gyrus; PCG: posterior cingulate gyrus. Amyloid positivity status was determined qualitatively and shown using dark orange if both visual readers identified the person as positive, and light orange if only one reader identified the visual positive.

Spread analyses suggested that amyloid deposition was most severe in the left posterior cingulate gyrus, where amyloid-positive individuals were most likely to cross standard cutoffs ( Fig. 3 ). Using a single Z-score cutoff as shown in Fig. 3A , the largest differences between negative and positive individuals were in the left frontal lobe, and analyses using region-specific cutoffs indicated that the presence of amyloid in the temporal and anterior cingulate cortex, while exhibiting relatively low Z-scores, was the most common. Of note, among individuals whose qualitative and quantitative results were non-concordant, quantitative measures of amyloid positivity were the lowest in the temporal and parietal lobes potentially suggesting that qualitative examinations are more difficult in these regions. By examining spread using the region-specific cutoffs determined in this study as shown in Fig. 3B , we found that though having relatively low Z-scores, amyloid was more often elevated in the temporal lobe and anterior cingulate gyri. Of note, when the temporal lobe and anterior cingulate gyrus were elevated, but other regions including the frontal and posterior cingulate gyrus were not sufficient to cross relatively higher Z-score cutoffs in those regions, qualitative results were often discordant from the quantitative results.

## Discussion

Amyloid PET provides a critical clinical neuroimaging tool in patients with possible AD. The purpose of this study was to evaluate whether qualitative adjudication of Aβ positivity in the clinical setting may be facilitated by relying on results from semi-quantitative Z-score analysis.

Amyloid PET allows for in vivo visualization of β-amyloid deposition, a hallmark pathologic change in AD that has been described to be the earliest neuroimaging predictor of future cognitive impairment in healthy elderly. 21 Available amyloid-targeting PET radiotracers include the research radiotracer [ ^11^ C]-Pittsburgh compound (PiB), as well as three FDA-approved [ ^18^ F]-labeled radiopharmaceuticals (florbetaben, florbetapir, and flutemetamol), all of which demonstrate high-affinity binding to cortical β-amyloid plaques. 22 23 While most of the early studies used [ ^11^ C]-PiB, the short half-life of ^11^ C limits its pragmatic clinical use. All three FDA-approved amyloid radiotracers have been shown to be acceptable clinical surrogates for PiB in the detection of Aβ and are widely used, with reported sensitivity and specificity of 90% or higher for the detection of increased cortical amyloid burden. In one study, cortical retention for each pair of tracers was strongly correlated, regardless of reference region (PiB-flutemetamol, ρ = 0.84–0.99; PiB-florbetapir, ρ = 0.83–0.97) and analysis method (ρ = 0.90–0.99). 24 25 Similarly, there was a strong association between PiB and florbetapir cortical retention ratios (Spearman ρ = 0.86–0.95). In another study, there was an excellent linear correlation between PiB and florbetaben global SUVR values ( *r* = 0.97, *p* < 0.0001) with similar effect sizes for distinguishing AD from control subjects for both radiotracers (Cohen's d = 3.3 for PiB and 3.0 for florbetaben). 26

Clinical interpretation is typically binary: “amyloid-positive” when there is evidence of cortical uptake and loss of gray–white matter differentiation versus “amyloid-negative” when there is non-specific white matter uptake and lack of cortical uptake resulting in preserved gray–white matter differentiation ( Figs. 1 and 2 ). 27 While amyloid PET can reliably detect β-amyloid deposition in vivo, the amyloid burden does not correlate with the degree of cognitive impairment, 21 and β-amyloid deposition can be seen in healthy older adults, adding an additional dimension to image interpretation in the clinical setting. 28 29 Therefore, a positive amyloid PET scan is necessary but insufficient to provide a positive diagnosis of AD, though a negative amyloid uptake has a good negative predictive value for AD. Among Medicare beneficiaries with MCI or dementia of uncertain etiology evaluated by specialists as part of the ACR-sponsored IDEAS study, amyloid PET was associated with changes in clinical management in over 60% of cases, while the etiologic diagnosis changed from AD to non-AD and vice versa in 35% of patients. 30 Furthermore, Aβ-amyloid PET imaging is widely used in patient selection and evaluation of treatment response and target engagement in disease-modifying clinical trials, and is expected to become a staple in the clinical management of AD patients, given recent regulatory approval of aducanumab, a high-affinity, fully human IgG1 monoclonal antibody against a conformational epitope found on Aβ in the brain. 14

Quantitative PET measurements have been reported to have a good correlation with visual interpretations, and standardized uptake value ratio (SUVR) and regional counts per reference count have been commonly used in quantitative analysis of amyloid PET. 31 Prior studies in the literature have assessed kinetic model-based approaches to quantify β-amyloid binding in the brain from dynamic PET data. For example, Becker et al 15 performed ^18^ F-florbetaben PET with concurrent multiple arterial sampling after tracer injection in AD subjects and controls. Regional brain-tissue time-activity curves for 90 min were analyzed by a one-tissue-compartment model and a two-tissue-compartment model (2TCM) with metabolite-corrected plasma data estimating the specific distribution volume (VS) and distribution volume ratio (DVR [2TCM]) and a multilinear reference tissue model estimating DVR (DVR [MRTM]) using the cerebellar cortex as the reference tissue. In their study, all β-amyloid-binding parameters (VS, DVR [2TCM], DVR [MRTM], and SUVR) were significantly increased in AD patients and excellent in discriminating between β-amyloid-positive and -negative scans. Furthermore, most amyloid PET cases can be easily classified as positive or negative on the bases of visual assessment; however, the findings are equivocal in approximately 10% of cases. 32 Because conventional mean cortical SUVR measures accumulation in both gray matter (GM) and white matter, it may misestimate amyloid deposits. Therefore, Ishii et al 16 developed a regional gray matter-dedicated SUVR (GMSUVR) system for amyloid PET images with three-dimensional (3D-MRI) and demonstrated its utility for discriminating between amyloid-positive and -negative subjects, even in cases where qualitatively, amyloid deposition was equivocal. In addition, neocortical atrophy typically present in elderly patients also reduces PET signal intensity, potentially affecting the diagnostic efficacy of β-amyloid PET data. Rullman et al 33 used partial-volume effect correction (PVEC), to adjust for atrophy bias, and demonstrated that PVEC improves quantitative analysis of ^18^ F-florbetaben PET scans. In clinical practice, careful attention should be paid to the selection of regions of interest (ROIs) by post-processing software as it may be suboptimal, particularly in small and adjacent regions such as the cingulate cortices, and in cases of advanced atrophy on structural imaging. 34 35 More recently, the Centiloid Project working group standardized quantitative amyloid imaging measures by scaling the outcome of each particular analysis method or tracer to a 0 to 100 scale, anchored by young controls (≤ 45 years) and typical AD patients, with units of this scale referred to as “Centiloids” for PiB PET and all three ^18^ F-labelled FDA-approved amyloid PET tracers. 36 37

Heavily quantitative approaches are cumbersome, require a trained quantitative analyst, and may not be feasible in a busy clinical practice. Additionally, not all clinical centers have access to the high-resolution 3D MRI sequences needed to help with this process. In a clinical setting, qualitative adjudication of Aβ positivity in clinical amyloid PET studies may be facilitated by relying on results from semi-quantitative Z-score analysis, which is of practical benefit, especially to trainees and inexperienced or infrequent readers. Therefore, we evaluated the correspondence between visual assessment and semiquantitative analysis in evaluating the cortical amyloid burden on clinical amyloid PET scans. Limitations of this study include its retrospective nature and lack of longitudinal follow-up. Additionally, only a single vendor post-processing algorithm was validated. Replication of these findings on other clinical PET software tools is needed prior to widespread implementation in routine clinical practice.

In conclusion, visual assessment and semiquantitative z-score analysis provide highly congruent results, thereby enhancing reader confidence and improving scan interpretation. This is particularly relevant given the recent emphasis on amyloid-targeting disease-modifying therapeutics, as they emerge from the research setting and enter clinical practice.
